# Prevalence of *Malassezia* species on the skin of HIV-seropositive patients

**DOI:** 10.1038/s41598-020-74133-6

**Published:** 2020-10-20

**Authors:** Paweł Krzyściak, Zofia Bakuła, Agnieszka Gniadek, Aleksander Garlicki, Mikołaj Tarnowski, Michał Wichowski, Tomasz Jagielski

**Affiliations:** 1 Faculty of Medicine, Chair of Microbiology, Department of Mycology, Jagiellonian University Medical College, Czysta 18, 31-121 Kraków, Poland; 2 Faculty of Biology, Institute of Microbiology, Department of Medical Microbiology, University of Warsaw, Miecznikowa 1, 02-096 Warsaw, Poland; 3 Faculty of Health Sciences, Institute of Nursing and Midwifery, Department of Nursing Management and Epidemiology Nursing, Jagiellonian University Medical College, Kraków, Poland; 4 Faculty of Medicine, Department of Infectious and Tropical Diseases, Jagiellonian University Medical College, Kraków, Poland; 5 Graduate of Faculty of Health Sciences, Jagiellonian University Medical College, Kraków, Poland

**Keywords:** Fungi, Fungal ecology, Fungal pathogenesis, Epidemiology, Symbiosis, Clinical microbiology, Clinical microbiology

## Abstract

*Malassezia* is a genus of lipophilic yeasts residing on the skin of warm-blooded animals. The correlation between specific species and their involvement in skin diseases has been well researched. However, only very few studies have investigated the distribution of *Malassezia* spp. on the healthy skin of patients infected with human immunodeficiency virus (HIV). The purpose of this work was to analyze whether the composition of *Malassezia* spp. isolated from the skin of the HIV-infected patients differs from that of healthy individuals. The study included a total of 96 subjects, who were divided into two equally sized groups: HIV-seropositive and HIV-seronegative. The specimens were collected from the subjects by swabbing four anatomical sites (face, chest, back, and scalp). Species were identified using phenotype-based methods, and the identification of strains isolated from the HIV-seropositive patients was confirmed by PCR sequencing of the rDNA cluster. *Malassezia* spp. were isolated from 33 (69%) HIV-seropositive patients and 38 (79%) healthy volunteers. It was found that men were much more likely to have their heads colonized with *Malassezia* spp. than women. The most prevalent species on the skin of both HIV-seropositive and HIV-seronegative individuals were *Malassezia sympodialis*, *M. globosa*, and *M. furfur*, albeit at different proportions in the two populations. The diversity of *Malassezia* spp. was the highest on the face of the HIV-seropositive patients (Shannon–Weiner Index H = 1.35) and lowest on the back of the healthy volunteers (H = 0.16). The phenotype- and molecular-based identification methods were congruent at 94.9%. It was observed a tendency that the HIV-seropositive patients had higher CD4+ cell counts, indicating higher colonization with *Malassezia* spp.

## Introduction

*Malassezia* (formerly *Pityrosporum*) is a genus of lipophilic basidiomycetous yeasts residing on the skin of humans and animals. Until recently, the Index Fungorum database has listed the names of only 23 valid species of this genus, including five that are doubtful, thus giving a total of 18 spp.^1,2^. However, some published studies suggested that distinct operational taxonomic units exist, and so the lists of species are not closed yet and the discovery of new species can be expected in the future^3^.⁠ Currently, the interactions between *Malassezia* spp. and the human host is intensively investigated, especially due to the fact that under certain conditions, these commensal fungi may behave as pathogens, causing skin disorders and even invasive infections.

The human skin is protected from the environment by the so-called skin surface lipid (SSL) film, which is a mixture made up of sebum and keratinocyte membrane lipids. Sebum, a product of the sebaceous glands, is released by holocrine secretion and coats both the skin and hairs. The human sebum consists of nonpolar lipids, triglycerides, wax esters, squalene, free long-chain fatty acids (containing up to 26 carbon atoms) which are either linear or branched and are mainly saturated or monounsaturated, and smaller amounts of cholesterol, esters, and diglycerides. The predominant fatty acid of sebum is sapienic acid (16:1, Δ6). The high percentage of long-chain fatty acids and polyterpenoid squalene is unique to human skin, but these are absent in the other human tissues and the sebum of nonhuman primates. Lipids of epidermal origin fill the spaces between the cells, like mortar, and constitute an insignificant fraction of the total extractable surface lipids on the areas that are rich in sebaceous glands. Keratinocytes produce lipids consisting of free fatty acids (FFAs), cholesterol, and ceramides in almost equal proportions^4^. As a whole, the fraction of SSL fatty acids is relatively scant in polyunsaturated fatty acids^5^.

The superficial layer of sebum, which is a residue of sweat, small quantities of intercellular lipids, and the components of natural moisturizing factors present on the skin surface, can differ based on sex and ethnicity and may reflect the underlying factors such as diet, hormonal levels, and enzyme expression^6^. The total amount of lipids is found to be apparently higher in the African American groups than in Caucasian Americans, regardless of age. The sebum levels of Asian Americans lie between that of the other two ethnic groups. The greatest difference among the ethnic groups was particularly observed in the fraction of wax esters. It has been reported that African American females in total have higher levels of wax esters than Caucasian American females^7^.

*Malassezia* living on humans are very specialized in utilizing human sebum triglycerides as food and are strictly lipid dependent. It was found that the genes related to fatty acid synthesis are absent in the genomes of *Malassezia globosa*, *Malassezia sympodialis*, and *Malassezia pachydermatis*. On the other hand, the genomes of *Malassezia* spp. are richer in lipase genes and lack carbohydrate-utilizing enzyme genes compared with the genomes of other sequenced fungi. The species also differ in terms of lipids assimilation: *Malassezia furfur* has the ability to assimilate palmitic acid, while assimilation defects are found in *M. globosa*, *M. sympodialis*, *M. pachydermatis*, and in the atypical variant of *M. furfur*^8^.

The difference between epidermal and sebum lipids can determine the metabolism of *Malassezia* and predispose individuals to colonization by specific species adopted to a different composition of lipid compounds. This explains the variations in the occurrence of particular species depending on ethnicity, sex, and skin location. The correlation between specific fungal species and the characteristics of host populations, such as sex, age, race, ethnicity, anatomical location, and most importantly, a particular skin disease, has been well researched^9–13^. The species distribution on the skin, as well as their worldwide distribution, varies between different *Malassezia*-related diseases^14^. For example, *M. sympodialis* is considered the most prevalent species in Europe, while *Malassezia restricta* and *M. globosa* are the most predominant in Asia. The difference in the species distribution may be related to not only the differences in geographic specificity but also the differences in the diagnostic methods used. Most of the European studies have used culture-based methods, whereas Asian studies have generally applied molecular-based methods. Moreover, some *Malassezia* spp. are slow-growing and more fastidious in culture, such as *M. restricta*, and these may be overgrown by a rapid-growing species, such as *M. sympodialis*^15^.

Fungi, as well as other microorganisms living on the human skin, come into contact with the host. The interactions between them can be mediated directly by specific pattern recognition receptors found on host cells and pathogen-associated molecular patterns present on fungal cell walls or indirectly via secreted factors^16^ and extracellular vesicles released from the fungal cells, which may assist the delivery of soluble mediators to the host cells^17–19^. *Malassezia* can produce metabolites such as indirubin and indolo[3,2‐b]carbazole, which are capable of stimulating the aryl hydrocarbon receptor (AhR) and can thereby modulate the functions of antigen-presenting cells, at least in vitro. AhR‐mediated signaling is appreciated as a major player in the physiology and pathology of the human skin which influences, for example, the skin’s responses to environmental toxins (i.e. dioxins) and bacterial stimuli^20^. *Malassezia*-derived metabolites, in particular indoles, can trigger AhR signaling, the activation of which can regulate skin homeostasis in manifold ways, including oxidation, epidermal barrier function, melanogenesis, and innate immunity⁠. Importantly, AhR has been implicated in type 17 immunity as it promotes Th17 differentiation and stimulates the production of IL-17A and related cytokines by Th17 cells and innate lymphoid cells (ILCs)^21^. IL-17 cytokines prevent the overgrowth of *Malassezia* on the murine skin^20^. A similar activity was described for IL-17 against other fungi, in particular *Candida albicans*, on the skin and mucosal surfaces^22^. However, no case has been reported with a genetic defect in the IL-17 signaling pathway manifesting as detectable overgrowth of *Malassezia* or leading to the development of *Malassezia*-associated skin disorders^20^.

Another immunological pathway that drives the polarization of CD4 + T cells into IL-17A-secreting effector cells is Card9-mediated signaling which couples the innate fungal recognition to the adaptive immune system^23^. It was found that in Card9-deficient mice and human, *Malassezia*-specific T helper cells belonged preferentially to the Th17 subset, and *Malassezia*-specific Th17 response was abolished^24^. Aside from αβ T cells, other IL-17A-producing cell types, such as γδ T cells and ILCs, were shown to respond swiftly to cytokine stimulation in an antigen-independent manner^25^. Intriguingly, Sparber et al. found that the Card9 pathway is not necessary for the induction of innate IL-17A by *Malassezia.* However, the underlying cause and the Card9-independent regulatory mechanisms of *Malassezia* are unclear so far^24^. Other classes of receptors, particularly those relevant to keratinocytes, which lack Syk/Card9-coupled C-type lectin receptors, are also implicated in the detection of *Malassezia* on the skin^20^.

The interaction between the *Malassezia* metabolites and the skin is not known. When applied topically, oleic acid, which is associated with the pathogenicity of dandruff and considered as an irritant causing dandruff‐like desquamation on the nonlesional scalp of dandruff patients, did not induce visible changes in nondandruff subjects^26^.

The skin of the human immunodeficiency virus (HIV)-infected patients is characterized by changes in both the composition of surface lipids, including SSL, and immune defense. Vidal et al. showed a significant increase in triglycerides and squalene and a decrease in FFAs in the HIV-positive patients regardless of the presence of seborrhoeic dermatitis (SD), compared to the HIV-negative patients with SD and healthy controls^27^. Osterle et al. found that the HIV-positive patients without SD had a significantly increased proportion of FFAs compared to the HIV-positive group with SD. Furthermore, in comparison to healthy participants, HIV patients had elevated levels of diglycerides, cholesterols, and FFAs, and lower levels of wax esters and squalene^28^.

HIV infection induces massive depletion of CD4 + T cells, mainly Th17, in the gastrointestinal tract. Th17 cells are lost very early during the course of HIV infection, which has been shown to correlate with bacterial translocation. Interestingly, even after successful antiretroviral therapy, these cells cannot be completely recovered from early destruction. Th17 and Th22 cells forming in response to antigens presented by activated dendritic cells from skin express homing receptors that promote their trafficking to the sites of inflammation in the skin. Specifically, skin-homing Th17 and Th22 cells contain chemokine receptors (CCR6+, CCR4+, CCR10+, CLA+), which direct their E selectin-dependent extravasation mediated by cutaneous lymphocyte antigen and migration along the gradients of chemokine ligands (CCL20, CCL22, and CCL27)^29^. As mentioned above, Th17 activation is a mechanism of defense against *Malassezia*, induced via secreted metabolites. Lack of these lymphocytes disturbs the immunological response of the skin, causing imbalance in skin microbial homeostasis, including the overgrowth of *Malassezia*.

The most prevalent *Malassezia*-associated skin infection is *pityriasis versicolor*, which presents as hyperpigmented or hypopigmented finely scaled macules or patches mainly in the neck, arms, and trunk. Another skin disease caused by *Malassezia* spp. is *folliculitis*, which typically manifests as pruritic, follicular papulopustular eruptions distributed on the back, chest, and upper limbs^30^. In other skin diseases including SD, atopic dermatitis (AD), and psoriasis (PS), the *Malassezia* yeasts exacerbate the symptoms and perpetuate the condition, probably due to the dysfunction of the skin barrier and exposure of the immune system to *Malassezia* antigens, which stimulate allergic inflammatory responses^31,32^. Such diseases are often found in HIV patients.

Central venous catheter-related *Malassezia* infections are more common in preterm neonates than adults. Systemic infections have been reported in the recipients of hematopoietic cell transplants, patients with underlying hematologic malignancies, cancer patients receiving monoclonal antibody therapy, and patients with other immunodeficiency states (e.g. solid organ transplantation, diabetes mellitus, prolonged glucocorticoid therapy, HIV). High temperature and humidity may facilitate the colonization of catheters, while lipid infusions might predispose individuals to both catheter colonization and infections. Additional risk factors of infections include low birthweight, severe comorbidities, and arterial catheterization for longer than 9 days. However, despite the presence of fungemia, disseminated fungal infection is uncommon^33^.

Most approaches focus on the role of *Malassezia* in skin diseases, especially severe SD, which is characteristic of HIV patients. So far, only very few studies have investigated the distribution of *Malassezia* spp. on the healthy skin of the HIV-infected patients^34–39^. This work analyzed whether the composition of *Malassezia* spp. isolated from the skin of the HIV-infected patients differs from that of healthy individuals.

## Results

### Demography and clinical details

The detailed demographic data of the HIV-seropositive patients and the control group are presented in Table 1. All the enrolled participants were of Caucasian origin.

**Table 1:** Patients’ demographic data.

		HIV (+)	HIV (−)	Statistics
Age	(Mean)	38.83 ± 9.38	38.85 ± 9.23	Student’s *t*-test, *p* = 0.991
	21–30	n = 9	n = 9	
	31–40	n = 21	n = 20	
	41–50	n = 13	n = 14	
	51–60	n = 4	n = 4	
	61 +	n = 1	n = 1	
Sex	Female	n = 9 (19%)	n = 13 (27%)	χ^2^-test, *p* = 0.331
	Male	n = 39 (81%)	n = 35 (73%)	
Place of residence	City	n = 41 (85%)	n = 37 (77%)	χ^2^-test, *p* = 0.296
	Village	n = 7 (15%)	n = 11 (23%)	
Education	Primary	6 (13%)	1 (2%)	Kolmogorov–Smirnov test, *p* = 1.000
	Vocational	8 (17%)	10 (21%)	
	Secondary	20 (42%)	16 (33%)	
	High	14 (29%)	21 (44%)	

Thirty-seven HIV-seropositive patients did not report taking any antifungals, while 11 patients declared using them (including four who probably used them due to coexistent oropharyngeal candidiasis). Six of the HIV-seropositive patients reported the presence of the following skin diseases: *pityriasis versicolor* (1), *folliculitis* (3), SD (1), and AD (1). The only changes observed were slight redness [11 (22.9%) HIV-seropositive patients and 13 (27.1%) healthy volunteers (*p* = 0.680)], slight hyperpigmentation (11 (22.9%) vs 9 (18.75%); *p* = 0.615), hypopigmentation (3 (6.25%) in both groups; *p* = 1.0000), and scaling (10 (20.8%) vs 6 (12.5%); *p* = 0.273). Given the benignity of the lesions, precluding diagnosis of any specific ailment, these patients were not excluded from the analysis. Thirty-four (34/48 or 71%) patients received antiviral therapy, with lopinavir/ritonavir being the most frequent combination.

### Mycological investigation

*Malassezia* spp. were isolated from 33 (69%) out of 48 HIV-seropositive patients (74% males and 44% females) and 38 (79%) out of 48 healthy subjects (83% males and 69% females). In eight HIV-seropositive patients (21.6%) who did not report the use of antifungal agents, *Malassezia* spp. were not found in the cultures derived from the specimens of any studied location. Likewise, *Malassezia* yeasts were not identified in seven (63.6%) out of 11 patients who used or were suspected of using antifungal agents (Fisher’s exact test, *p* = 0.0219).

The most colonized body sites were the chest and shoulders, while the least colonized was the head. No differences were observed in the frequency of isolation depending on the HIV status in each of the studied locations (Mantel–Haenszel test, *p* = 0.508) (Table 2).

**Table 2:** Frequency of *Malassezia* spp. isolation from the skin of the HIV-seropositive patients and healthy volunteers according to body site.

Body site	Culture	HIV(+)	HIV(−)	Total	*P *(χ^2^-test)
Head	(+)	11 (22.9%)	6 (12.5%)	17 (17.7%)	0.1813
	(−)	37 (77.1%)	42 (87.5%)	79 (82.3%)	
Chest	(+)	21 (43.8%)	30 (62.5%)	51 (53.1%)	0.0657
	(−)	27 (56.2%)	18 (37.5%)	45 (46.9%)	
Back	(+)	22 (45.8%)	26 (54.2%)	48 (50.0%)	0.4142
	(−)	26 (54.2%)	22 (45.8%)	48 (50.0%)	
Face	(+)	14 (29.2%)	13 (27.1%)	27 (28.1%)	0.8204
	(−)	34 (70.8%)	35 (72.9%)	69 (71.9%)	

**Figure 1:**
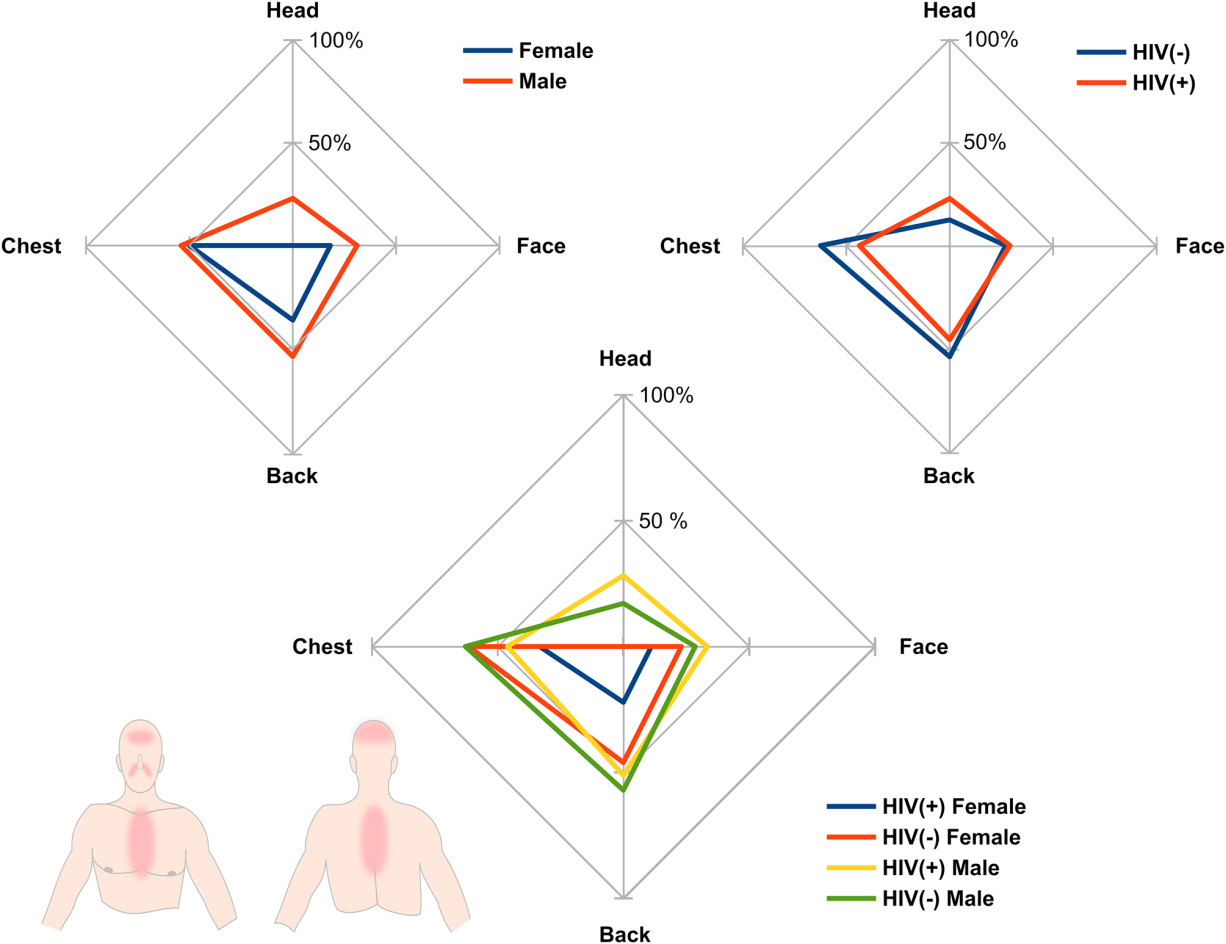
The occurrence of *Malassezia* on different body sites based on sex (first chart), HIV status (second chart), and the combination of all variables (third chart).

Men were much more likely to have their heads colonized with *Malassezia* spp. compared to women (16% vs 0%, respectively). This finding was statistically significant in the case of the control group (Fisher’s exact test, *p* = 0.022) and the two groups combined (Fisher’s exact test, *p* = 0.010). When the results from the two groups were analyzed together, the back of men was found to be more frequently colonized with *Malassezia* spp. compared to women (*χ*^2^-test, *p* = 0.015; Fig. 1).

### Prevalence of *Malassezia* spp.

**Figure 2:**
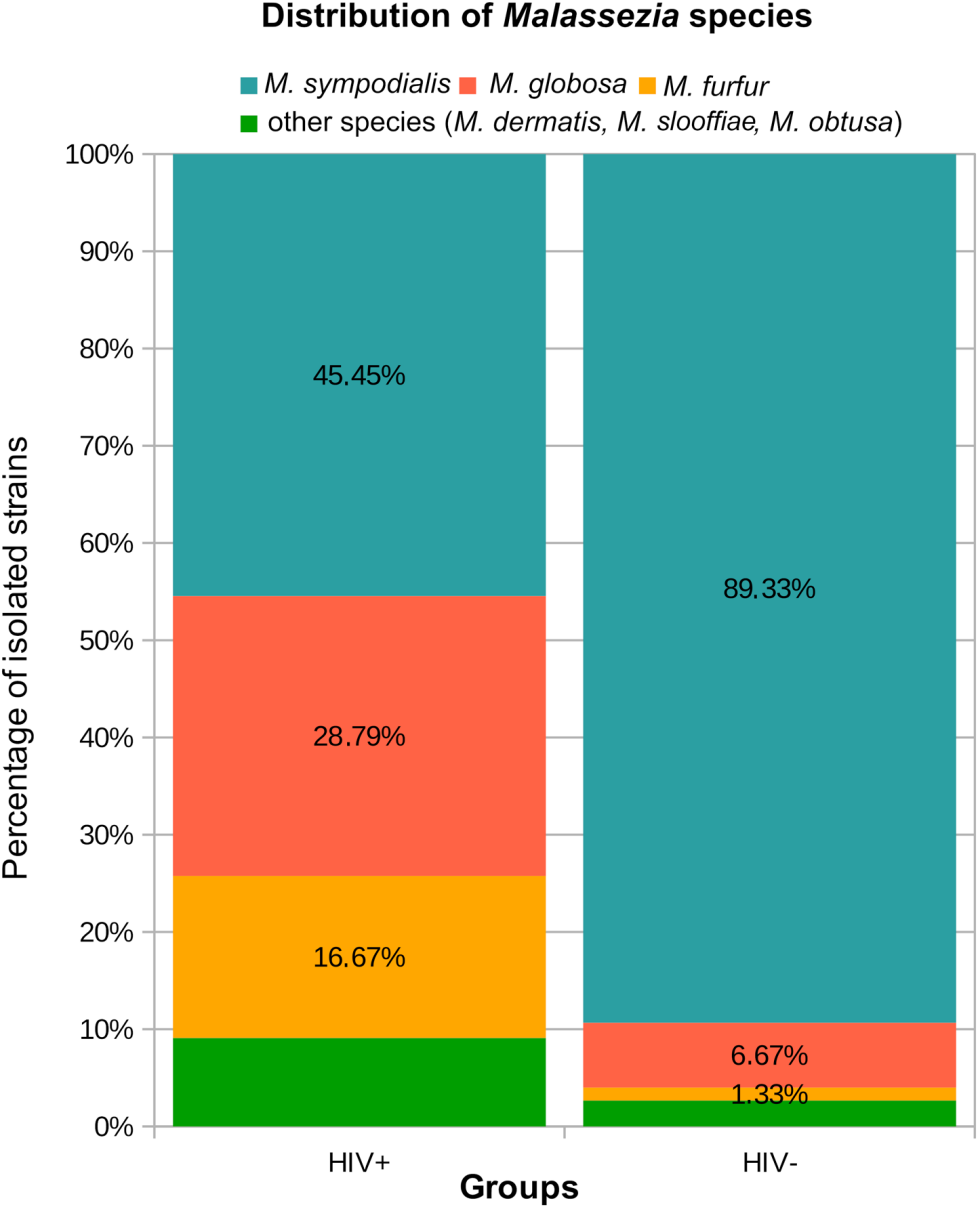
Distribution of *Malassezia* spp. among strains isolated from the HIV-positive and HIV-negative patients.

The two most prevalent *Malassezia* species on the skin of the HIV-seropositive and HIV-seronegative individuals were *M. sympodialis* and *M. globosa*; however, their frequencies differed considerably in the two populations (45.5% vs 89.3% and 28.8% vs 6.7%, respectively). *Malassezia furfur* was isolated from 16.7% of the HIV-seropositive patients, and only from 1.3% of healthy volunteers. Three spp., namely *Malassezia slooffiae*, *M. restricta*, and *Malassezia obtusa*, were isolated only from the HIV-seropositive patients, but at a low frequency of 3% (*M. slooffiae*, *M. restricta*) or 1.5% (*M. obtusa*). The detailed data are shown in Table 3 and Fig. 2.

**Table 3:** Prevalence of *Malassezia* spp. (results from PCR-sequencing identification in the brackets) with adopted Shannon–Weiner Index of *Malassezia* biodiversity in the investigated locations (calculated for species identification confirmed by PCR).

	Group	Head	Chest	Back	Face	Total	Percentage
*M. dermatis*	HIV(+)	0	1	0	0	1	1.5
	HIV(−)	0	1	1	0	2	2.7
*M. furfur*	HIV(+)	3	2 (3)^a^	2 (3)^a^	4	11	16.7
	HIV(−)	0	1	0	0	1	1.5
*M. globosa*	HIV(+)	2	9 (8)^a^	4	4	19	28.8
	HIV(−)	2	2	0	1	5	6.7
*M. obtusa*	HIV(+)	0	0	1 (0)^a^	0	1	1.5
	HIV(−)	0	0	0	0	0	
*M. restricta*	HIV(+)	0	0	0	2	2	3
	HIV(−)	0	0	0	0	0	
*M. slooffiae*	HIV(+)	0	0	2	0	2	3
	HIV(−)	0	0	0	0	0	
*M. sympodialis*	HIV(+)	5	8	13 (13)^a^	4	30	45.4
	HIV(−)	4	26	25	12	67	89.3
Total	HIV(+)	10	20	22	14	66	100
	HIV(−)	6	30	26	13	75	100
Person with no growth of *Malassezia*	HIV(+)	37	27	26	34	15	
	HIV(−)	42	18	22	35	10	
Shannon–Wiener Index	HIV(+)	1.03	1.17	1.03	1.35	1.08	
	HIV(−)	0.63	0.53	0.16	0.27	0.43	

^a^One strain of *M. globosa* (168) and one strain of *M. sympodialis* (150 II) were verified as *M. furfur,* while one strain of *M. obtusa* (123) was verified as *M. sympodialis.*

The biodiversity of *Malassezia* spp. was the highest on the face of the HIV-seropositive patients (H = 1.35), while it was the lowest on the back of healthy volunteers (H = 0.16; Table 3).

### Results of species verification by PCR methods

Of the 65 strains isolated from the HIV-seropositive patients, molecular identification was successful in 59 (90.8%) cases. Thirty (50.8%) out of 59 isolates had a 99–100% similarity with the sequence of *M. sympodialis*, 17 (28.8%) showed 98–100% sequence identity with *M. globosa*, and 10 (16.9%) showed 99–100% sequence similarity with *M. furfur.* One (1.7%) out of 59 isolates was identified as *Malassezia dermatis* (99% sequence similarity) and another one (1.7%) as *M. slooffiae* (100% sequence similarity) (Supplementary Table). Three (5.1%) out of 59 isolates produced discrepant results between phenotypic and molecular identification. These included two *M. furfur* isolates, which were phenotypically identified as *M. sympodialis* and *M. globosa*, and one *M. sympodialis* isolate, which upon biochemical profiling was identified as *M. obtusa*. Thus, the concordance rate between the two methods was calculated at 94.9% (56/59 cases).

### *Malassezia* spp. and CD4+ lymphocyte level

**Figure 3:**
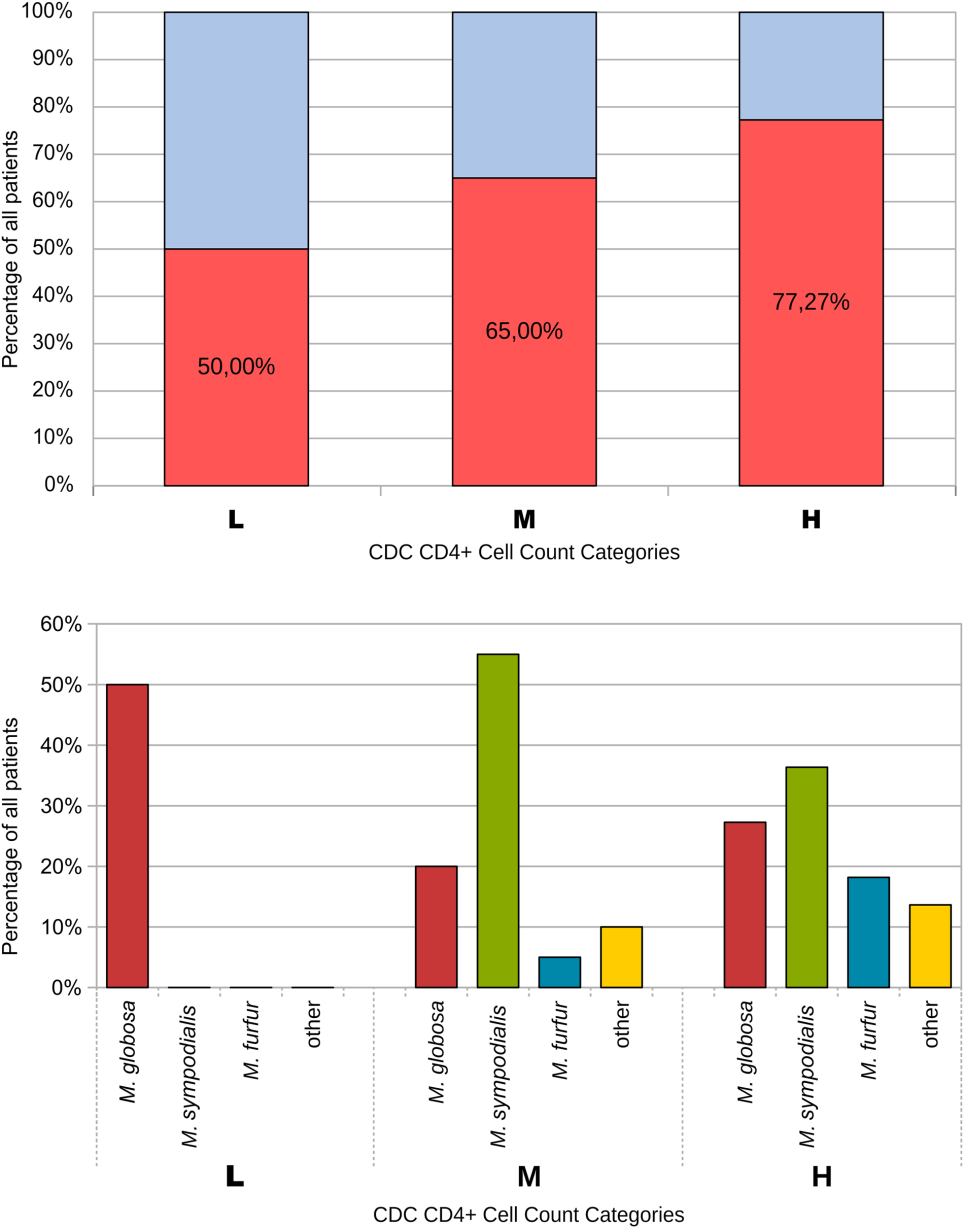
Relation of *Malassezia* colonization and CD4+ counts (top); distribution of main species among CD + groups (bottom). L < 200 cells/µl, M 200–499 cells/µl, and H > 500 cells/µl.

Among the HIV-seropositive patients, the number of CD4+ cells was ≥ 500/cm^−3^ in 22 (45.8%), 200–499 cells/cm^−3^ in 13 (27.1%), and < 200 cells/cm^−3^ in six (12.5%). Patients with a higher CD4+ cell count showed higher colonization with *Malassezia* spp.; however, the difference was statistically insignificant (Fig. 3).

## Discussion

Numerous studies have focused on the role of *Malassezia* yeasts in the pathogenesis of SD, AD, and PS^40^, but only a few have analyzed the importance of *Malassezia* as a part of natural microbiota on the healthy skin in the context of HIV status. There are two approaches to determining the presence of microbes in a given niche. The first is methods consisting of the isolation of microorganisms on culture media, followed by their identification (by morphological, biochemical, or by nucleic acid analysis or protein analysis methods). The second method consists in searching for the nucleic acid corresponding to taxa directly in the sample and does not require the isolation of living microorganisms. The limitation of the first one is the different survival of microorganisms outside the host organism, which means that some species may not be detected at all. Also, the proportions of species may be disturbed in favor of species growing faster on the media. In the genomics methods, the results appear to be more reliable. However, finding of fungal DNA does not necessarily equate the presence of viable cells.

As described in the Introduction, HIV patients differ from the noninfected individuals in SSL composition and immunological defense. It is therefore expected that the healthy skin of HIV patients should have different *Malassezia* spp. independent of skin diseases. The main finding of this study was the differences in the composition of *Malassezia* species between the populations examined. Generally, it was more diverse in the HIV-seropositive group than in healthy subjects. Regardless of the presence of *Malassezia*, HIV infection is regarded as a predisposing factor for SD, and plenty of studies have investigated the role of *Malassezia* in SD. However, because of the lack of broad lipidomic, genomic, and metabolomic studies, we are not sure if there is confusion between cause and effect.

In this study, we found no differences in the frequency of *Malassezia* isolation, depending on the HIV status. Håkansson et al. quantified *Malassezia* cultures retrieved from 12 HIV-seropositive and 12 HIV-seronegative homosexuals and showed that the mean number of *Malassezia*/cm^2^, the mean serum antibody titers against the yeasts, and the occurrence of cutaneous disorders did not differ significantly between the two studied groups^38^. Likewise, no differences were found between the HIV-seropositive and control groups by de Vroey and Song, who evaluated samples from the forehead and back of subjects and by Wikler et al. who compared samples from the chest and back^35,36^.

di Silverio et al. found that the abundance of *Malassezia* spp. was greater on the skin of HIV-seropositive than healthy individuals when they evaluated the number of fungal elements in microscopic smears from scalp, forehead, nose, and axillae. However, no differences were found when smears from the chest, groin, and back were compared between the two patient groups^37^. Pechere and Saurat compared the density of *Malassezia* yeasts on the forehead of 40 HIV-seronegative and 38 HIV-seropositive people with clinically normal skin and found that it was much higher in the latter group^41^.

In the present study, the most prevalent species, with abundant growth, was found to be *M. sympodialis* which was observed in 89.3% of healthy volunteers and 45.4% of the HIV-seropositive patients. This species was mainly recovered from the chest and back of healthy subjects, with an isolation rate of 52–54%. The next two most common species were *M. globosa* and *M. furfur*, which were found at a higher frequency in the HIV-seropositive individuals (i.e. 28.8% vs 16.7% and 6.7% vs 1.5%, respectively). A previous study on the prevalence of *Malassezia* spp. in the Polish population, showed the dominance of *M. sympodialis* with single *M. globosa*, *M. slooffiae,* and *M. restricta*. However, the results of that study, as well as ours, may have been influenced by the use of the culture method^11^. In the same temperate climate and similar latitude, a Canadian study was performed by Gupta and Kohli using the contact plate method. They found a very congruent distribution of *Malassezia* spp. in the HIV-seronegative population, where the isolation rates of *M. sympodialis*, *M. globosa*, and *M. furfur* were estimated at 56.9%, 31.8%, and 6.1%, respectively^10^.

In Europe*, M. sympodialis* was found as the predominant species on healthy skin, especially on the trunk, in Bosnian and Herzegovinian populations^42^. This species was also most commonly found on the skin of healthy people or the healthy skin of dermatological patients in Spain^43^ and Sweden^13^. In a Portuguese study which was based on the contact plate method and used adhesive tape for sample collection on the forearm and matched site, *M. sympodialis* was shown to be strongly predominant, followed by single *M. restricta* and *M. globosa*^44^. In the study of Aspiroz et al. (1999), the most abundantly isolated species in healthy Spanish people was *M. globosa*, yet *M. sympodialis* was predominant on the back^44^.

All the abovementioned studies are based on culture methods. In one study based on direct microbiome analysis, *M. restricta* (47%) was dominant on the side of the nose of healthy Swiss individuals, and *M. sympodialis* (25%) was the second most frequent species^45^. Furthermore, in the study on Bosnian and Herzegovinan populations, *M. restricta* was the most prevalent species on healthy scalp, while *M. furfur *and *M. sympodialis *were more frequent in the cultures derived from healthy trunk skin^42^.

*Malassezia* colonization in Asians is very distinct, but two species are dominant: *M. restricta* and *M. globosa*. It was found that *M. sympodialis* was less frequent even in the culture-dependent studies*.* In a Japanese study conducted using both culture and nonculture methods, *M. restricta* was the predominant species on the face of men, *M. globosa* and *M. dermatis* on the upper trunk of men, and *M. globosa* and *M. sympodialis* on the upper trunk of women, but there were seasonal variations observed in the results^9^. Another large Japanese study based on direct PCR method conducted on 770 healthy subjects showed that the predominant species was *M. restricta*, followed by *M. globosa*, and the findings varied depending on age and sex^46^ Similar results were found in a study from South Korea, where the dominant species were *M. restricta* (55%) and *M. globosa* (22.5%), while *M. sympodialis* was isolated only in 10% of patients, mainly from the chest^47^. In healthy Singaporean, the rate of positive yeast culture was 87.5% for the side of the nose, with *M. globosa* (56.8%), *M. furfur* (48.6%), and *M. restricta* (40.5%) being the dominant species. From the scalp samples, two species *M. globosa* (47.6%) and *M. restricta* (23.8%) were isolated^45^. Honnavar et al. analyzed the sociodemographic characteristics and spectrum of *Malassezia* spp. in individuals with and without SD/dandruff in India and found that in rural populations *M. globosa* was predominant on the scalp, followed by *M. furfur* and *M. restricta* (in nearly one-third of the population, no growth of *Malassezia* was detected). In the urban population, the scalp was colonized mostly by *M. restricta* (33%) and *M. globosa* (26%), while one-fourth showed no growth of *Malassezia*. In the nasolabial area, *M. globosa* (14%) was dominant, but three-fourths of the participants showed no growth of *Malassezia*^48^. Skin scraping analysis (culture method) showed that *M. furfur* (85%) and *M. globosa* (10%) were dominant in the thorax of 222 healthy Chinese subjects^49^.

Analysis of data on the prevalence of *Malassezia* spp. in different ethnic groups is hard not only due to discrepancies in the sampling methodologies and approaches to obtain information about species (culture vs omics methodology) but also due to numerous additional factors. The primary factor is the difference in the sebum composition of individuals with different origins. Six fatty acids were identified that were significantly different in quantity between African and Caucasian Americans—synthesized naturally in the skin, as the 14:0, 16:1, n10, and 18:1, n9 fatty acids; acquired exclusively from the diet, as the iso-18:2 fatty acid; and others may be a product of bacterial metabolism, as the 15:0 or 17:1 fatty acids—which would indicate microbiota differences on the surface of the skin between these ethnic groups^7^. Further influencers of *Malassezia* factors are the place of living (urban or rural area), hygiene habits (washing/oiling the body and hair), use of cosmetics and their composition, climatic differences even within a given country (dry in the mountain area or humid on the coast), clothing composition (natural or artificial), etc.^46,48,50^. Moreover, the spectrum and frequency of *Malassezia* spp. in healthy individuals have been shown to differ according to sex, location on the skin, and sampling season; species diversity and isolation rate have been found to be the highest in samples collected from the upper trunk of men during summer^9,51^. For this reason, although all the study participants were Caucasians, we tried to make our groups very similar in terms of sex, age, place of living, etc.

Differences in the isolated *Malassezia* spp. were observed among HIV-seropositive patients, with respect to their CD4+ counts. In general, the lower was the CD4+ count, the lower was the recovery rate of *Malassezia* spp. Although the observed correlation was not statistically significant, some studies have described a similar observation. In a study from Indonesia, the density of *Malassezia* cells showed no significant relationship with the CD4+ lymphocyte count, albeit the numbers supported our observations, with lower *Malassezia* CFU counts seen in patients with low CD4+ levels^52^. The most recent study of Moreno-Coutino et al. showed very similar results (rate of *Malassezia* isolation from patients with CD4+ counts > 500, 200–499, and < 200 cells/mm^−3^ was 60%, 27%, and 13%, respectively). Interestingly, patients with SD did not show this correlation in their study^39^. A possible explanation of this association is the disequilibrium of concurrent microbiota of skin due to the extensive treatment of viral, bacterial, and fungal dermatoses in neutropenic patients.

Another explanation is antifungal prophylaxis in immunosuppressed patients. In this study, only one patient with a low CD4+ count (i.e. < 200 cells/cm^−3^) declared using fluconazole. In addition, the use of antiseptic cosmetics and personal care products could potentially affect the results.

The proportion of *Malassezia* spp. among HIV patients with a high level of CD4+ lymphocytes was not similar to the proportion among healthy controls. This fact suggests that immunological response has no direct influence on *Malassezia* diversity.

According to Gupta and Kohli^10^, *Malassezia* spp. colonized the skin of 80–90% of healthy volunteers who were aged 15 years or older. In their study, the rate of *Malassezia* colonization (growth from at least one anatomical site) was 79% among healthy volunteers and 69% among the HIV-seropositive patients. In our study, we found a relatively low rate of colonization in comparison to others. In particular, we did not isolate any *Malassezia* strain from the scalp of women. This could be explained by the wide use of antidandruff shampoos with antimicrobial compounds in Poland. We did not control this factor, and the data collected from subjects were difficult to interpret because many patients did not pay attention to the type of shampoo they used. However, we noticed that 40% of participants declared using antidandruff shampoo, but there was no difference in the occurrence of *Malassezia* among groups (data not shown).

The underrepresentation of *M. restricta* could be explained by the slower growth and formation of small colonies on Leeming Notman agar, Oil-Potato Dextrose agar, and mDA compared to other species^53^. Studies based on molecular methods in different populations frequently have shown *M. restricta* as the most abundant *Malassezia* species on healthy and diseased human skin. Some other factors, including those related to population, may also play a role in this relationship.

The results of phenotypic and molecular identification were discordant in approximately 5% of cases. The reason for this might be misidentification with the use of phenotypic tests. Molecular-based methods serve as a more accurate and reliable means of species identification, since phenotypic tests might reveal only important diversity within *Malassezia* spp.^54,55^.

A limitation of the study was that only isolates recovered from the HIV-seropositive patients were subjected to confirmatory speciation using the molecular typing method. This was because isolates from healthy subjects were not preserved and were unavailable for subsequent molecular studies. However, since we observed a high concordance of results between the phenotype-based and molecular-based methods used for species identification among the HIV-positive patients (95%), and the species composition determined only by conventional methods, as in the case of the HIV-seronegative group, seems to be highly accurate.

Another limitation of the study was that the prevalence of other fungi and bacteria was not investigated in the evaluated skin samples. Indeed, the exploration of the entire skin mycobiome of the study subjects, which is achievable using metagenomic sequencing, would add more information for a better understanding of any potential shifts in the skin microflora associated with chronic inflammatory conditions, such as HIV infection.

In conclusion, the examined HIV-seropositive and HIV-seronegative individuals showed the same three core *Malassezia* spp. and rank-frequency distribution. In the former group, however, the species spectrum was wider to some extent. To better recognize this relationship, further studies with more patients are needed.

## Materials and methods

### Patient recruitment procedure

The study analyzed a total of 96 individuals. This included 48 HIV-seropositive patients, who were treated at the Clinic of Infectious Diseases, Department of Gastroenterology, Hepatology and Infectious Diseases, Jagiellonian University in Kraków, and 48 healthy (HIV-seronegative) volunteers (control group).

Written informed consent statements obtained from all the HIV-infected patients and healthy volunteers, of both sexes and over the age of 18 years, who were included in the study. Patients with conditions that would not allow collecting material or who did not give the written consent for participation were excluded. The specimens were collected from 16.03.2011 to 27.04.2012. The participants were also surveyed during sample collection.

The study followed the Declaration of Helsinki guidelines (2008) and was approved by the Bioethics Committee of the Jagiellonian University (No. KBET/33/B/2011, dated 28.04.2011).

### *Malassezia* isolation

Specimens were obtained from four anatomical sites: face (nasolabial wrinkles and forehead), chest (breastbone), back (interscapular region), and scalp. They were collected by rubbing 10–15 times with viscose swabs moistened with sterile saline. The samples were delivered to the laboratory within 2 h, where they were immediately inoculated onto modified Dixon’s agar (mDA) medium (malt extract 36 g, mycological peptone 10 g, ox bile 20 g, Tween 40 10 cm^−3^, glycerol 2 cm^−3^, oleic acid 2 cm^−3^, chloramphenicol 0.5 g, and agar 15 g/1000 cm^−3^ distilled water) and cultured in a humid chamber at 32 °C for 2 weeks and checked at 7 and 14 days.

### Species identification

#### Biochemical methods

**Figure 4:**
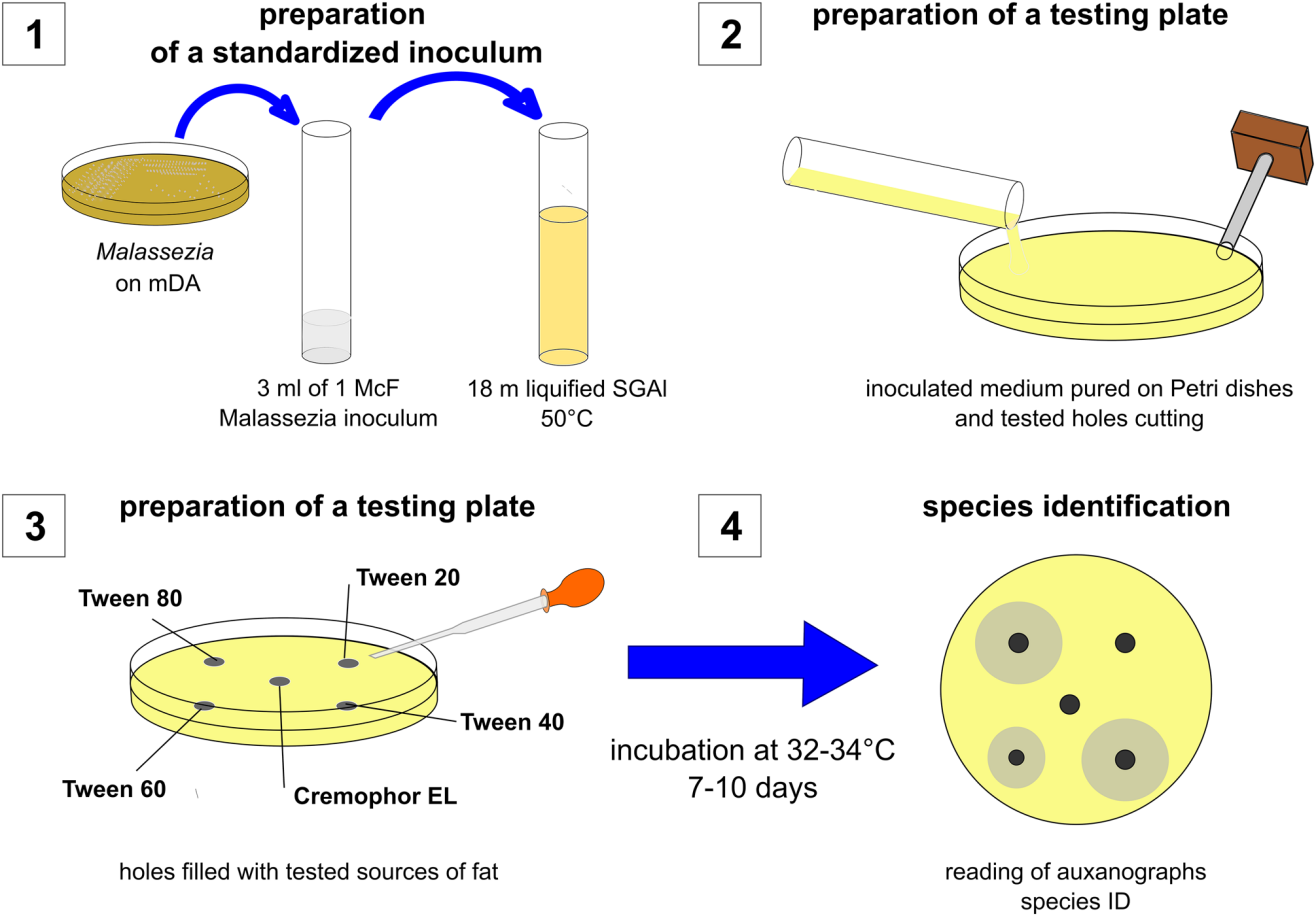
Biochemical identification *of Malassezia*. Three milliliters of standardized cell suspensions were prepared by adding an actively growing colony to sterile water and adjusted to 1 on the McFarland scale. Next, the suspensions were added to the liquefied Sabouraud Glucose Agar medium and cooled to approximately 50 °C to solidify (1). In the solid medium, five holes of 2 mm were prepared (2) and filled with lipid compounds (Tween and Cremophor EL) (3). The prepared plates were incubated for 7–10 days at 32–34 °C and read visually (4).

Species were first identified using phenotypic methods, which involved the examination of macro- and micromorphologies and determination of biochemical profiles based on the organisms’ requirement for exogenous lipid for their growth, catalase production, esculin hydrolysis, and assimilation of Cremophor EL and Tween. All the phenotypic tests were carried out and interpreted essentially as described elsewhere^56^ (Fig. 4).

Strains isolated from the HIV-seropositive patients, after biochemical identification, were inoculated into cryo-tube with modified Dixon broth which was supplemented with glycerol and gradually cooled: firstly in a refrigerator to 4 °C, then in a freezer to − 22 °C, and finally stored at − 80 °C for further analysis^57^.

#### Molecular methods

For strains isolated from the HIV-seropositive patients, the identification of phenotype-based species was confirmed by molecular typing. Briefly, DNA was extracted from yeast cultures on mDA medium, using Genomic Mini AX Yeast kit (A&A Biotechnology, Poland) following the manufacturer’s instructions.

Molecular identification was performed by sequence analysis of a part of the rDNA cluster, comprising the internal transcribed spacer (ITS) regions ITS1 and ITS2, a complete 5.8S rRNA gene, and partial regions of the 18S and 26S rRNA genes. For this purpose, primers V9 (5′-TGCGTTGATTACGTCCCTGC-3′) and RLR3R (5′-GGTCCGTGTTTCAAGAC-3′) were used. PCR amplification, sequencing, sequence alignment, and mapping were carried out as described previously^11^. In short, the PCR mixtures with a total volume of 25 µl, containing approximately 20 ng of genomic DNA each, were prepared using TopTaq Master Mix kit (Qiagen, Germany) following the vendor’s protocol. After initial denaturation at 94 °C for 5 min, the reaction mixtures were run through 30 cycles at 94 °C for 45 s, 56 °C for 30 s, and 72 °C for 1.5 min, and a final step at 72 °C for 10 min. Amplicons were sequenced either directly with the same primers as those used for the amplification, or when difficult-to-read sequences were obtained, after cloning into the pGEM-T Easy Vector system (Promega, USA). Consensus sequences were assembled with ChromasPro ver. 1.7.1 (Technelysium, Australia) and searched against the GenBank database of the National Center for Biotechnology Information (NCBI) using the BLASTN algorithm (https://blast.ncbi.nlm.nih.gov/). For species identification, distance scores of up to 2.00% (98% match) were used as a proxy, and the species showing the closest match was considered as correctly identified.

All the determined nucleotide sequences were deposited in the GenBank database under the accession numbers listed in Supplementary Table.

### CD4+ level

The level of CD4+ lymphocytes in HIV patients was calculated during routine diagnostics at the Clinic of Infectious Diseases using flow cytometry methods with FACS Canto II (Becton Dickinson).

### Data collection and statistical analysis

Statistical analysis was carried out using language and environment for statistical computing and graphics R (GNU General Public License). Test results with *p* < 0.05 were considered statistically significant^58^.

The biodiversity of *Malassezia* spp. was determined using Shannon–Wiener Diversity Index [H] as follows^59^:

$$H = - \sum\limits_{i = 1}^{S} {p_{i} \log p_{i} }$$

where pi = ni/Ni (ni is the number of persons colonized by a particular *Malassezia* sp. and Ni is the number of persons colonized by any *Malassezia* sp.).

### Ethics approval

Bioethics Committee of the Jagiellonian University (No. KBET/33/B/2011, dated 28.04.2011).

### Consent to participate

Each patient signed the written consent.

## Supplementary information

Supplementary Table.

## References

[1] Kirk, P. M. Index Fungorum : Search Page. *Index Fungorum* https://www.indexfungorum.org/Names/Names.asp (2020).

[2] Lorch JM (2018). *Malassezia vespertilionis* sp. Nov.: a new cold-tolerant species of yeast isolated from bats. Persoonia.

[3] Soares RC, Zani MB, Arruda ACBB, de Arruda LHF, Paulino LC (2015). Malassezia intra-specific diversity and potentially new species in the skin microbiota from brazilian healthy subjects and seborrheic dermatitis patients. PLoS ONE.

[4] Pappas A (2009). Epidermal surface lipids. Dermatoendocrinology.

[5] De Luca C, Valacchi G (2010). Surface lipids as multifunctional mediators of skin responses to environmental stimuli. Med. Inflamm..

[6] Shetage SS, Traynor MJ, Brown MB, Chilcott RP (2018). Sebomic identification of sex- and ethnicity-specific variations in residual skin surface components (RSSC) for bio-monitoring or forensic applications. Lipids Health Dis..

[7] Pappas A, Fantasia J, Chen T (2013). Age and ethnic variations in sebaceous lipids. Dermatoendocrinology.

[8] Triana S (2017). Lipid metabolic versatility in *Malassezia* spp. yeasts studied through metabolic modeling. Front. Microbiol..

[9] Akaza N (2010). Cutaneous *Malassezia *microbiota of healthy subjects differ by sex, body part and season. J. Dermatol..

[10] Gupta AK, Kohli Y (2004). Prevalence of *Malassezia *species on various body sites in clinically healthy subjects representing different age groups. Med. Mycol..

[11] Jagielski T (2014). Distribution of *Malassezia* species on the skin of patients with atopic dermatitis, psoriasis, and healthy volunteers assessed by conventional and molecular identification methods. BMC Dermatol..

[12] Rup E, Skóra M, Krzyściak P, Macura AB (2011). Distribution of *Malassezia *species in patients with atopic dermatitis: quality assessment. Postepy Dermatol. Alergol..

[13] Sandström Falk MH (2005). The prevalence of *Malassezia* yeasts in patients with atopic dermatitis, seborrhoeic dermatitis and healthy controls. Acta Derm. Venereol..

[14] Grice EA, Dawson TL (2017). Host-microbe interactions: *Malassezia *and human skin. Curr. Opin. Microbiol..

[15] Saunte DML, Gaitanis G, Hay RJ (2020). Malassezia-associated skin diseases, the use of diagnostics and treatment. Front. Cell. Infect. Microbiol..

[16] Velegraki A, Cafarchia C, Gaitanis G, Iatta R, Boekhout T (2015). Malassezia infections in humans and animals: pathophysiology, detection, and treatment. PLoS Pathog..

[17] Johansson HJ (2018). Extracellular nanovesicles released from the commensal yeast *Malassezia sympodialis* are enriched in allergens and interact with cells in human skin. Sci. Rep..

[18] Zhang YJ (2019). Extracellular vesicles derived from *Malassezia furfur* stimulate IL-6 production in keratinocytes as demonstrated in in vitro and in vivo models. J. Dermatol. Sci..

[19] Vallhov H, Johansson C, Veerman RE, Scheynius A (2020). Extracellular vesicles released from the skin commensal yeast malassezia sympodialis activate human primary keratinocytes. Front. Cell. Infect. Microbiol..

[20] Sparber F, Ruchti F, LeibundGut-Landmann S (2020). Host immunity to malassezia in health and disease. Front. Cell. Infect. Microbiol..

[21] Furue M, Takahara M, Nakahara T, Uchi H (2014). Role of AhR/ARNT system in skin homeostasis. Arch. Dermatol. Res..

[22] Sparber F, Leibundgut-Landmann S (2019). Interleukin-17 in antifungal immunity. Pathogens.

[23] LeibundGut-Landmann S (2007). Syk- and CARD9-dependent coupling of innate immunity to the induction of T helper cells that produce interleukin 17. Nat. Immunol..

[24] Sparber F (2019). The skin commensal yeast Malassezia triggers a type 17 response that coordinates anti-fungal immunity and exacerbates skin inflammation. Cell Host Microbe.

[25] Cua DJ, Tato CM (2010). Innate IL-17-producing cells: the sentinels of the immune system. Nat. Rev. Immunol..

[26] DeAngelis YM (2005). Three etiologic facets of dandruff and seborrheic dermatitis: Malassezia fungi, sebaceous lipids, and individual sensitivity. J. Investig. Dermatol. Symp. Proc..

[27] Vidal C (1990). Seborrheic dermatitis and HIV infection: qualitative analysis of skin surface lipids in men seropositive and seronegative for HIV. J. Am. Acad. Dermatol..

[28] Ostlere LS (1996). Skin surface lipids in HIV-positive patients with and without seborrheic dermatitis. Int. J. Dermatol..

[29] Bixler SL, Mattapallil JJ (2013). Loss and dysregulation of Th17 cells during HIV infection. Clin. Dev. Immunol..

[30] Boekhout T, Mayser P, Guého-Kellermann E, Velegraki A (2010). Malassezia and the Skin.

[31] Theelen B (2018). Malassezia ecology, pathophysiology, and treatment. Med. Mycol..

[32] Gaitanis G, Velegraki A, Mayser P, Bassukas ID (2013). Skin diseases associated with *Malassezia* yeasts: facts and controversies. Clin. Dermatol..

[33] Spelman, D. & Morrissey, M. B. *Invasive Malassezia Infections: UpToDate*. https://www.uptodate.com/contents/invasive-malassezia-infections (2020).

[34] Pechère M, Saurat JH (1997). Malassezia yeast density in HIV-positive individuals. Br. J. Dermatol..

[35] Wikler JR, Nieboer C, Willemze R (1992). Quantitative skin cultures of Pityrosporum yeasts in patients seropositive for the human immunodeficiency virus with and without seborrheic dermatitis. J. Am. Acad. Dermatol..

[36] De Vroey C, Song MBT-M, Bossche H (1990). Dermatophytes and Pityrosporum in AIDS patients ecology and epidemiology. Mycoses in AIDS Patients.

[37] Di Silverio A (1991). Prevalence of dermatophytes and yeasts (*Candida* spp., Malassezia furfur) in HIV patients: a study of former drug addicts. Mycopathologia.

[38] Håkansson C, Faergemann J, Löwhagen GB (1988). Studies on the lipophilic yeast Pityrosporum ovale in HIV-seropositive and HIV-seronegative homosexual men. Acta Derm. Venereol..

[39] Moreno-Coutiño G (2019). Isolation of Malassezia spp. in HIV-positive patients with and without seborrheic dermatitis. An. Bras. Dermatol..

[40] Ramos-E-Silva M, Lima CMO, Schechtman RC, Trope BM, Carneiro S (2010). Superficial mycoses in immunodepressed patients (AIDS). Clin. Dermatol..

[41] Pechere M, Saurat JH (1997). Malassezia yeast density in HIV-positive individuals. Br. J. Dermatol..

[42] Prohic A, Jovovic Sadikovic T, Krupalija-Fazlic M, Kuskunovic-Vlahovljak S (2016). Malassezia species in healthy skin and in dermatological conditions. Int. J. Dermatol..

[43] Crespo Erchiga V, Ojeda Martos A, Vera Casano A, Crespo Erchiga A, Sanchez Rajardo F (1999). Aislamiento e identification de Malassezia spp. en pitiriasis versicolor, dermatitis seorreica y piel sana. Rev. Iberoamer. Micol..

[44] Aspiroz C, Moreno LA, Rezusta A, Rubio C (1999). Differentiation of three biotypes of Malassezia species on human normal skin. Correspondence with *M. globosa**M. sympodialis* and *M. restricta*. Mycopathologia.

[45] Leong C (2019). Geographical and ethnic differences influence culturable commensal yeast diversity on healthy skin. Front. Microbiol..

[46] Sugita T (2010). Quantitative analysis of the cutaneous Malassezia microbiota in 770 healthy Japanese by age and gender using a real-time PCR assay. Med. Mycol..

[47] Lee YW, Yim SM, Lim SH, Choe YB, Ahn KJ (2006). Quantitative investigation on the distribution of Malassezia species on healthy human skin in Korea. Mycoses.

[48] Honnavar P (2020). Sociodemographic characteristics and spectrum of Malassezia species in individuals with and without seborrhoeic dermatitis/dandruff: a comparison of residents of the urban and rural populations. Med. Mycol..

[49] Li W (2020). Molecular epidemiology, in vitro susceptibility and exoenzyme screening of Malassezia clinical isolates. J. Med. Microbiol..

[50] Piérard-Franchimont C, Uhoda E, Loussouarn G, Saint-Léger D, Piérard GE (2003). Effect of residence time on the efficacy of antidandruff shampoos. Int. J. Cosmet. Sci..

[51] Findley K (2013). Topographic diversity of fungal and bacterial communities in human skin. Nature.

[52] Panjaitan E, Retno Pudjiati S, Sri Siswati A (2015). Low CD4+ T cell counts are not risk factor for Malassezia species infection in HIV/AIDS patients. J. Thee Med. Sci..

[53] Kaneko T (2005). Vital growth factors of *Malassezia *species on modified CHROMagar Candida. Med. Mycol..

[54] Puig L, Bragulat MR, Castellá G, Cabañes FJ (2018). Phenotypic and genetic diversity of Malassezia furfur from domestic and zoo animals. Med. Mycol..

[55] Hurtado-Suárez A (2016). Phenotypic characterization of canine *Malassezia* spp., isolates. Rev. MVZ Cordoba.

[56] Kaneko T (2007). Revised culture-based system for identification of *Malassezia* species. J. Clin. Microbiol..

[57] Crespo MJ, Abarca ML, Cabañes FJ (2000). Evaluation of different preservation and storage methods for *Malassezia* spp. J. Clin. Microbiol..

[58] R Core Team. *R: a language and environment for statistical computing* (R Foundation for Statistical Computing, Vienna, Austria, 2020). https://www.R-project.org

[59] Chiarucci A, Bacaro G, Scheiner SM (2011). Old and new challenges in using species diversity for assessing biodiversity. Philos. Trans. R. Soc. B.

